# Condom use and sexuality communication with adults: a study among high school students in South Africa and Tanzania

**DOI:** 10.1186/1471-2458-13-874

**Published:** 2013-09-23

**Authors:** Francis Sande Namisi, Leif Edvard Aarø, Sylvia Kaaya, Hans E Onya, Annegreet Wubs, Catherine Mathews

**Affiliations:** 1 African Medical and Research Foundation (AMREF), P.O. Box 27691–00506, Nairobi, Kenya; 2 Department of Health Promotion and Development, Faculty of Psychology, University of Bergen, P.O. Box 7807, N-5020, Bergen, Norway; 3 Division of Mental Health, Norwegian Institute of Public Health, Oslo, Norway; 4 Department of Psychiatry, Muhimbili University of Health and Allied Sciences, P.O. Box 65001, Dar es Salaam, Tanzania; 5 Department of Medical Sciences, Public Health and Health Promotion, School of Health Sciences, University of Limpopo, Turfloop Campus, Private Bag X1106, Sovenga 0727, South Africa; 6 Health Systems Research Unit, Medical Research Council, P.O. Box 19070, Tygerberg, 7505 Cape Town, South Africa; 7 Adolescent Health Research Unit, Department of Psychiatry and Mental Health, University of Cape Town, University Private Bag, Rondebosch, 7700 Cape Town, South Africa

**Keywords:** Adolescents, Parents, Condom use, Communication, South Africa, Tanzania

## Abstract

**Background:**

Fostering adolescents’ communication on sexuality issues with their parents and other significant adults is often assumed to be an important component of intervention programmes aimed at promoting healthy adolescent sexual practices. However, there are few studies describing the relationship between such communication and sexual practices, particularly in sub-Saharan Africa. This study examined the relationships between adolescents’ communication with significant adults and their condom use in three sites in this region.

**Methods:**

Data stem from a multi-site randomized controlled trial of a school-based HIV prevention intervention implemented in Cape Town and Mankweng, South Africa and Dar es Salaam, Tanzania. Only data from comparison schools were used. The design is therefore a prospective panel study with three waves of data collections. Data were collected in 2004 from 6,251 participants in 40 schools. Associations between adolescents’ communication with adults about sexuality issues and their use of condoms were analysed cross-sectionally using analysis of variance, as well as prospectively using multiple ordinal logistic regression analysis.

**Results:**

Cross-sectional analyses showed that consistent condom users had significantly higher mean scores on communication (across topics and communication partners) than both occasional users and never-users, who had the lowest scores. After controlling for condom use at the first data collection occasion in each model as well as for possible confounders, communication scores significantly predicted consistent condom use prospectively in all three ordinal logistic regression models (Model *R*^*2*^ = .23 to .31).

**Conclusion:**

The findings are consistent with the assertion that communication on sexuality issues between adolescents and significant adults results in safer sexual practices, as reflected by condom use, among in-school adolescents. The associations between communication variables and condom use might have been stronger if we had measured additional aspects of communication such as whether or not it was initiated by the adolescents themselves, the quality of advice provided by adults, and if it took place in a context of positive adult-adolescent interaction. Studies with experimental designs are needed in order to provide stronger evidence of causality.

## Background

Whereas the rate of new HIV infections is falling in a number of countries in sub-Sahara Africa, the region has continued to experience a heavy burden of people living with HIV (22.5 million in 2009) [1]. This translates into 68% of the global total HIV burden. Among the approximately 5 million young people (age 15–24) who lived with AIDS worldwide in 2008, 76% were in sub-Saharan Africa [2]. However, it is clear that there are sub-regional variations in the prevalence. For instance in 2009, southern Africa was reported as the most severely affected with an estimated 11.3 million people living with HIV, with South Africa’s epidemic alone accounting for almost 50% [1].

During recent years there has been an observable decline in HIV incidence within sub-Saharan Africa and in particular within the eastern Africa sub-region. For example, the United Republic of Tanzania between 2004 and 2008 recorded a decline in HIV incidence of 3.4 per 1000 persons [1]. The evidence also suggests that there is a decline of varying levels in Botswana, South Africa, Zambia, and Zimbabwe. It is worth noting that the countries reporting these significant declines in prevalence also concurrently observed significant changes in sexual behaviour among both young women and men [1]. While there is remarkable progress in averting new HIV infections worldwide as well as reducing the number of annual AIDS related deaths, the number of people living with HIV is still on the increase [1]. Towards this end, HIV remains a public health priority in sub-Saharan Africa where heterosexual sex accounts for the largest proportion of the new infections. There is a need to develop and implement more effective behavioural interventions that complement the scaled-up Anti-Retroviral Treatment public health programmes. Behavioural interventions aimed at reducing new infections, as well as complementing treatment programmes, are a priority, particularly among the adolescent population.

### Parent–child communication in the context of parent–child interaction

The focus of the present study is on the relationship between adolescents’ interpersonal communication with adults (parents, other adult family members and teachers) and behaviour (adolescent condom use). According to Collins & Laursen [3] no aspect of adolescent development has received more attention from researchers than parent-adolescent relationship and influences. According to Stattin & Kerr [4] aspects of parents’ behaviour such as parental monitoring and control have been assumed to foster mature and responsible social behaviour among children and adolescents. Studies have shown, however, that control is not enough. Collins and Laursen [3] add that also interpersonal warmth, accepting attitudes, bidirectional communication, and an emphasis on training social responsibility and concern for the impact of one’s action on others are important factors contributing to socially responsible behaviour among adolescents. Beveridge and Berg [5] describe the adolescent-parent interaction as a process of collaboration and maintain that “… parents and adolescents who engage in friendly autonomous processes that display and encourage independence, and who provide appropriate levels of control characterized by warmth and guidance have adolescents who experience positive adaptation”.

Darling and Steinberg [6] have conceptualized parenting styles as a context within which socialization occurs and emphasized that the effects of parenting practices can only be understood in the context of parenting styles. Consistent with Baumrind [7], they use the term ‘authoritative’ to describe parenting styles characterized by reciprocity of communication and use of reasoning and explanation. Other important features of authoritative parenting are warmth and autonomy granting. In families where the parenting style can be described as authoritative, communication skills are generally assumed to be higher than in families where parenting is authoritarian or permissive [3].

Also Kerr and Statin [8] have maintained that parents’ tracking and surveillance efforts are not as effective as previously thought. They even claim that vigilant surveillance and tracking might be linked to some forms of poor adjustment. The key to understanding behavioural outcomes of the parent-adolescent relationship is not to be found in the behaviour of parents per se, but in the interaction between parents and their children. Adolescents are themselves active agents in the process through which parents keep track of where their children spend time, with whom they are, and what they are doing. They are, in fact, parents’ primary source of information about adolescents’ whereabouts and activities. Kerr and Stattin [8] have suggested that children’s spontaneous disclosure of information is a key factor in understanding young people’s adjustment. The focus of the present study is therefore not on how specific parental practices are related to condom use as a responsible social behaviour, but rather on how level of interpersonal communication between adolescents and three categories of adults (parents, other adult family members and teachers) is related to such behaviour.

Communication with parents and with other adult family members is generally assumed to contribute to healthy sexual practices among adolescents [9]. However, as revealed in the literature review below, there is paucity of studies from sub-Saharan Africa that could throw light on this. Furthermore, previous studies carried out in sub-Saharan Africa or elsewhere do not provide consistent evidence of a positive association between adolescents’ communication on sexuality issues with parents and other significant adults and consistent condom use. In the present study we will use data from three sites in sub-Saharan Africa in order to examine associations between adolescents’ communication on sexuality issues with significant adults and their use of condoms cross-sectionally as well as prospectively.

The results of the present study should be seen in the context of previous research on the relation between communication between parents, other adults and adolescents on one side and condom use among adolescents on the other. We will distinguish between observational studies and experimental (intervention) studies, and we will cover studies from sub-Saharan African as well as studies from other regions of the world.

### Previous observational studies

In some cross-sectional studies from the United States significant positive associations between parent-offspring communication on sexuality and condom use have been reported [10-14]. In one of these studies, however, significant associations were found among those who reported high levels of monitoring only [11]. In another study significant associations were found only if parents were open, skilled and comfortable in having those discussions [14]. Miller and associates [13] found maternal condom discussions prior to sexual debut to be associated with greater condom use. Cross-sectional analyses of data from one nation-wide U.S. prospective panel study showed unexpectedly, however, that discussion with parents of the risks and potential negative consequences of sexual activity was associated with greater likelihood of condom non-use [15].

Reporting from a cross-sectional study among young adults in Thailand, Rasamimari et al. [16] found no association between communication with parents on dating, sex related issues, alcohol and drugs and use of condoms. Wang, Hsu & Wang [17], from a study among male high school students in Taiwan, reported no significant association between parental communication on contraception and intentions to use contraceptives. Wight and associates [18] carried out a prospective longitudinal study among secondary school students in Scotland and reported that the degree of comfort in talking with parents about sex had only limited prospective association with condom use.

In two cross-sectional studies from sub-Saharan Africa positive associations between communication with significant adults and condom use among adolescent were reported [19,20]. In one study no significant positive associations were found [21]. Some studies have not reported associations with condom use specifically, but used broader measures of use of contraceptives. Biddlecom, Awusabo-Asare & Akinrinola [22] carried out a study among unmarried 15–19 year olds in Burkina Faso, Ghana, Malawi, and Uganda. Parent–child sexuality communication was positively and significantly associated with contraceptive use for Ghanaian females and Ugandan females and males only. Among eight associations between information about contraceptive methods from parents (or parental figure) and having used a contraceptive method at last sex, none proved significant.

In summary, there is at present not much strong evidence from observational studies for a protective effect of parent-offspring communication on sexuality with regard to condom use. This conclusion is consistent with a recent review by Markham and associates [23].

### Intervention studies

Evaluations of interventions involving parents as sex educators or promoting parent-offspring communication on issues related to sexual behaviour have shown positive effects on condom use or use of contraceptives among adolescents and young adults in sub-Saharan Africa [24,25] as well as elsewhere [26-28]. In these studies promotion of parent-offspring communication on sexuality issues was only one out of several intervention elements and activities. Specific effects of the parent communication component are therefore not well documented.

Few studies of the relationship between adolescents’ communication with significant adults on sexuality issues and their condom use have been carried out in sub-Saharan Africa, and all observational studies have been cross-sectional. Cross-sectional studies cannot throw much light on causality. Although prospective longitudinal studies have their shortcomings, they represent an important step towards stronger evidence for causal relationships.

### The present study

The present study investigates whether adolescents’ sexuality communication with significant adults (parents, other adult family members, and teachers) about sexuality related to three topics, HIV/AIDS, abstinence, and use of condoms, is associated with condom use among adolescents cross-sectionally as well as prospectively.

## Methods

### Study context

This study was part of a multi-site cluster randomized controlled trial of an AIDS prevention intervention (the SATZ project) [29]. Three African research institutions were involved: University of Cape Town (South Africa), University of Limpopo (Polokwane, South Africa), and Muhimbili University of Health and Allied Sciences (Dar es Salaam, Tanzania). In this publication the institutions will be referred to as project partners (Cape Town, Limpopo and Muhimbili), while study sites refers to the districts where the studies were carried out (Cape Town, Mankweng and Dar es Salaam). The aim of the analyses presented in this publication was to examine statistical relationships cross sectionally as well as prospectively in the absence of program interventions, and thus only control group schools were included in the current analysis. The study included 12 schools in Dar es Salaam, 13 in Cape Town and 15 in Mankweng.

### Participants

The study included students in grade 8 in South Africa (Cape Town and Mankweng) and grades 5 and 6 in Tanzania (Dar es Salaam). The students at the three sites were aged 12–15 at the time of study. Baseline data collection (T1) took place in February and March 2004, and two follow-up waves of data collections (T2 and T3) were conducted approximately 6 and 12 months thereafter. Altogether, the study included 6,251 students (47.8% male) across the three locations and all three data collections, including 2714 from Cape Town, 1317 from Mankweng, and 2220 from Dar es Salaam. Broken down by wave of data collection, there were 5,878 participants at T1, 5,776 at T2, and 4,823 at T3. While data were collected from 6,251 students, not all of them were present at all data collections, hence the discrepancy between the overall N and those at each data collection occasion.

For the initial analyses (descriptive statistics and intercorrelations among communication sumscores) all students with valid answers were included. In the analyses of prospective prediction of condom use, only those students who reported having had first sexual intercourse (anal or vaginal) were included.

### Procedures

Local teams of trained researchers and research assistants carried out data collection in class. Teachers were not present during these activities. The lead researcher in each study site was responsible for training the local research assistants and for supervising and ensuring quality data collection and entry before transmission to the coordination office at Bergen University in Norway. In Cape Town, palmtop computers were used for data collection, while in Mankweng, and Dar es Salaam, printed questionnaires were used. The Mankweng researchers read the questions aloud to the class while students filled in the questionnaire. Although there was some variation in data collection procedures across sites, analyses of scale properties of data from the T1 data collection did not reveal any systematic differences in dimensionality and reliability of scales. Confidentiality was ensured during the entire data collection exercise in all three study sites.

### Measures

The project partners collectively developed the final international English version of the questionnaire [29]. The translations into other languages (Afrikaans, Xhosa, Sepedi, Swahili) were conducted locally at each of the three study sites. Trained local research teams executed back translation, examination, pilot testing and revisions of the instruments [30]. The final questionnaire consisted of 155 items, including socio-demographic factors, sexual behaviours, psychosocial variables (such as attitudes, social norms, self-efficacy, intentions), and interpersonal communication variables.

### Condom use

A consistent condom user was defined as one who answered (i) yes when asked whether s/he and the partner used a condom the first time they had sexual intercourse, (ii) yes when asked if s/he and the partner always used a condom whenever they had sex and (iii) no to the question of whether s/he had ever had sex with the partner without using a condom. Never-users were those who answered ‘no’ to these two questions: ‘The first time you had sexual intercourse, did you/your partner use a condom?’ and ‘Have you and your partner ever used a condom during sex?’ The remaining participants were defined as occasional users of condoms. This procedure was followed for all three data collection occasions.

### Communication variables

Participants were asked how often each of the following categories of adults, parents/guardians, other adult family members, and teachers, talked with them on each of three topics: HIV/AIDS, abstinence, and condoms. Response categories were scored on a 5-point scale ranging from “never” (1), “hardly ever” (2), “sometimes” (3), “a lot” (4) to “all the time” (5). We computed mean scores (scale 1–5) of the three reproductive health items for communication with parents/guardians, other family members (OFMs) and teachers separately. In addition, we computed an overall communication mean score “total communication” across all three reproductive health topics and the three categories of adult communication partners. This was done for each of the three data collection occasions.

We also constructed a separate set of communication variables (based on the same set of items) by producing one sumscore for each communication topic, adding across communication partners. This was also done for each data collection occasion. Tables for analyses involving these sumscores are not presented.

### Demographic and other variables

The socio-demographic predictors included were the participant’s age (at T1), gender (male, female), study site (Cape Town, Mankweng, Dar es Salaam), and socio-economic status (SES) (at the most recent data collection for each model). The SES index, described in an earlier publication [31], was a meanscore (sumscore divided by number of items) based on three indicators: (i) number of assets in family (TV, bicycle etc.), (ii) number of people sleeping in the same room (log natural transformed and reversed) and (iii) a subjective report about the material wealth of the family (five categories from “not enough money for food” to “enough for luxury items”). All these three indicators were standardized (mean = 0.00 and standard deviation = 1.00) before the meanscore calculation, and the final SES meanscore was also standardized. Among the socio-demographic variables, age and SES were treated as metric predictors in the logistic regression analyses.

Perceived access to condoms was measured with three items: (i) If you needed a condom, how easy or difficult would it be for you to obtain one?, (ii) I would be able to go to a clinic to fetch condoms, (iii) I would be able to go to a pharmacy or a shop to buy condoms. All items had five response categories ranging from ‘very easy’ to ‘very difficult’ (first item) or ‘I strongly agree’ to ‘I strongly disagree’ (last two items). Items were recoded in order for high scores to reflect good access to condoms. Being victim of partner violence was measured with five items: (a) ‘Have you ever had a boyfriend /girlfriend who beats you up?’ (b) ‘Has a girlfriend/boyfriend ever used a knife or other weapon against you?’ A dichotomous violence exposure variable was constructed. Yes on at least one of the five statements was coded as 1 (one). No on all of them was coded as 0 (zero).

### Data analysis

We used SPSS (Version 20) in our statistical analyses. Simple descriptive statistics (means and standard deviations) and correlations were used for describing properties of the communication sumscores. Cronbach’s alpha was used to estimate internal consistency of scales. Associations between condom use and communication sumscores were analyzed using general linear models (GLM), and prospective prediction of condom use was analyzed with multiple ordinal logistic regression. Complex designs statistics were used in order to control for the cluster effect with schools as the clustering unit.

### Ethics

Participation in the study was voluntary, and the students gave assent (active consent). Parents or caregivers were informed, and their children were allowed to participate in the study unless their parents refused (passive consent). For all three sites, procedures for handling questionnaires and computerizing of data which ensured confidentiality were followed. All cases in the data set were de-identified (so that individuals could not be linked with their responses) at the start of the data analysis process.

Ethical clearance was provided by the Regional Committee for Medical Research Ethics for Western Norway and by relevant ethics committees in the three African study sites: The Human Research Ethics Committee, Health Sciences Faculty, University of Cape Town; The Senate Research and Publication Committee at Muhimbili University of Health and Allied Sciences (Dar es Salaam); MEDUNSA Research Ethics Committee, University of Limpopo. The study was also approved by the Data Protection Officer for Research, of the Norwegian Social Science Data Services.

## Results

### Descriptive results

Table 1 shows descriptive statistics for the communication sumscore variables at an individual communication partner level as well as that of the three communication partners combined, for each of the three data collection occasions. The mean scores range from 2.31 to 2.69 and standard deviations from 1.08 to 1.30. The Cronbach’s alpha coefficients of the sumscore for each communication partner separately range from .83 to .89, while the alpha values for the global communication sumscores range from .91 to .93. The highest cross-sectional correlations among sumscores were obtained for communication with parents and communication with other family members (.74-.79 across measurement occasions). Correlations between communication with teachers and the other two sumscores were generally lower (.52-.62) (tables not shown).

**Table 1:** Communication with parents/guardians, other adult family members and teachers – meanscore descriptives (scale range for all meanscores is 1–5)

**Communication variables**	**Data collection**	**Number of items**	**n**	**M**	**SD**	**n**	**Alpha**
Communication with parents/guardians	First	3	5086	2.36	1.23	4913	.83
	Second	3	5300	2.39	1.24	5151	.85
	Third	3	4518	2.40	1.24	4377	.87
Communication with other adult family members	First	3	5050	2.31	1.24	4896	.86
	Second	3	5272	2.33	1.24	5127	.87
	Third	3	4474	2.32	1.25	4346	.89
Communication with teachers	First	3	5075	2.64	1.27	4942	.87
	Second	3	5358	2.69	1.30	5219	.88
	Third	3	4486	2.67	1.30	4361	.89
Communication with all three partners combined	First	9	5180	2.43	1.08	4617	.91
	Second	9	5433	2.47	1.10	4875	.92
	Third	9	4575	2.45	1.11	4108	.93

Number of observations is lower for alpha, since alpha is calculated only when all items have valid values.

### Bivariate associations of communication variables across three time points

Table 2 shows correlations across data collection occasions for each sumscore. All correlations are positive and statistically significant (p<.001). The strongest associations were observed between variables measured at the second and third data collections (*r* = .45-.52) and between the first and second data collections (*r* = .48-.52). For the longest time span (between first and third data collections) the correlations were generally slightly lower (*r =*.40-.47). This pattern of differences among associations was consistent across all the three individual communication partner sumscores as well as for the total sumscores.

**Table 2:** Correlation among communication sumscores (meanscores) across the three data collection occasions

Communication partner (n*)	**Data collection occasion**			
		**1**	**2**	**3**
Parents/guardians (n=3716-4458)	1. First	1.00		
	2. Second	.49	1.00	
	3. Third	.42	.48	1.00
Other adult family members (n=3663-4409)	1. First	1.00		
	2. Second	.48	1.00	
	3. Third	.40	.45	1.00
Teachers (n=3693-4491)	1. First	1.00		
	2. Second	.48	1.00	
	3. Third	.43	.49	1.00
Communication with all three partners (n=3828-4626)	1. First	1.00		
	2. Second	.52	1.00	
	3. Third	.47	.52	1.00

All correlations significant at *p<* 0.001.

* Ranges of n are reported for the off-diagonal correlation coefficients only.

Number of cases (n) for diagonals are identical to those listed in the first n column in Table 1.

### Cross-sectional associations between communication variables and condom use

On each of the three data collection occasions (T1, T2 and T3), all sumscores (communication with parents/guardians, other family members, teachers and all three partners combined) were significantly associated with condom use at that occasion (p < .05) (Table 3). Highest mean scores were obtained for those who reported to be consistent condom users and lowest mean scores were observed among never-users. The mean scores for those who reported some, but not consistent condom use, were in most cases (one exception only) located between those of consistent condom user and never-user groups.

**Table 3:** Communication on HIV/AIDS, abstinence and condoms with the three partners by condom use adjusted for site, age, and gender and cluster effect (school)

	**Communication with**^**1**^**:**	**Never users**		**Occasional users**		**Consistent users**		***p<***
		**n**	**Mean**	**n**	**Mean**	**n**	**Mean**	
**Time 1**	Parents/guardians	364	2.37	610	2.53	125	2.90	.001
	Other adult family members	362	2.31	612	2.67	125	2.98	.001
	Teachers	365	2.73	609	2.86	125	3.18	.05
	All the three partners (Total)	371	2.48	618	2.68	126	3.02	.001
**Time 2**	Parents/guardians	337	2.36	651	2.46	142	2.73	.05
	Other adult family members	335	2.31	643	2.51	142	2.74	.001
	Teachers	336	2.74	644	2.74	145	3.20	.05
	All the three partners (Total)	344	2.49	659	2.58	145	2.90	.01
**Time 3**	Parents/guardians	271	2.30	538	2.58	152	2.80	.001
	Other adult family members	268	2.31	534	2.56	150	2.89	.001
	Teachers	266	2.78	533	2.84	150	3.12	.05
	All the three partners (Total)	274	2.46	541	2.66	151	2.93	.001

T1, T2 and T3 – first, second and third data collections.

^1^ Each score represents the respondents’ average across all three communication topic areas.

### Prospective prediction of consistent condom use

To investigate the predictive power of communication on consistent condom use versus never-use prospectively, we performed multiple ordinal logistic regression analyses with these predictors: site, gender, age, condom use (at the first of the relevant pair of measurement occasions), communication on sexuality (also at the first relevant occasion), socio-economic status, perceived access to condoms, and exposure to violence (Table 4). For the last three variables, we used data from the last of the two relevant measurement occasions. In each of the logistic regression models for the three time frames (T1-T2, T1-T3 and T2-T2), the dependent variable had three levels; never-use, occasional use and consistent use. The explained variances (Nagelkerke’s pseudo *R*^*2*^) when predicting condom use prospectively were 31%, 23% and 28% (T1-T2, T1-T3 and T2-T3).

**Table 4:** Condom use (never-use = 0; occasional use = 1; consistent use = 2) by communication at previous data collection occasion and other predictors

**Time**	**Predictors**	**n**	**OR (95% CI)**	***p<***
**T1 – T2**	**Site**			.05
	Dar es Salaam	170	1.00	-
	Mankweng	358	1.75 (1.11 - 2.76)	.05
	Cape Town	490	1.83 (1.14 - 2.94)	.05
	**Gender**			.05
	Male	747	1.00	
	Female	171	1.82 (1.03 - 3.24)	
	**Condom use at T1**			.001
	Never-users	218	1.00	-
	Occasional users	368	5.84 (3.00 - 11.37)	.001
	Consistent users	88	36.49 (14.40 - 92.42)	.001
	Sexually inactive	344	6.35 (3.43 - 11.78)	.001
	**Victims of violence at T2**			n.s.
	No	615	1.00	
	Yes	403	1.05 (.84 - 1.33)	
	**Age**	-	1.12 (1.03 - 1.21)	.05
	**Access condoms at T2**	-	1.28 (1.14 - 1.45)	.001
	**SES at T2**	-	1.14 (0.97 - 1.34)	n.s.
	**Communication at T1**	-	1.22 (1.08 - 1.37)	.01
Nagelkerke’s pseudo R^2^ =.313				
**T1 –T3**	**Site**			.001
	Dar es Salaam	170	1.00	-
	Mankweng	298	2.56 (1.67 - 3.91)	.001
	Cape Town	379	2.55 (1.72 -.3.77	.001
	**Gender**			.001
	Male	598	1.00	
	Female	249	1.96 (1.40 - 2.75)	
	**Condom use at T1**			.001
	Never-users	149	1.00	-
	Occasional users	249	3.20 (1.97 - 5.20)	.001
	Consistent users	56	8.34 (4.12 - 16.85)	.001
	Sexually inactive	393	3.30 (2.04 - 5.31)	.001
	**Victims of violence at T3**			.n.s.
	No	507	1.00	
	Yes	340	1.02 (0.80 - 1.29)	
	**Age**	-	0.99 (.90 - 1.09)	n.s.
	**Access condoms at T3**	-	1.38 (1.18 - 1.61)	.001
	**SES at T3**	-	1.34 (1.15 - 1.56)	.001
	**Communication at T1**	-	1.21 (1.04 - 1.41)	.05
Nagelkerke’s pseudo R^2^ =.226				
**T2 - T3**	**Site**			.001
	Dar es Salaam	202	1.00	-
	Mankweng	296	2.71 (1.81 - 4.07)	.001
	Cape Town	401	3.09 (2.10 - 4.55)	.001
	**Gender**			.001
	Male	643	1.00	
	Female	256	1.79 (1.34 - 2.39)	
	**Condom use at T2**			.001
	Never-users	158	1.00	-
	Occasional users	329	2.80 (1.75 – 4.46)	.001
	Consistent users	78	15.73 (6.78 - 36.52)	.001
	Missing	334	3.03 (1.70 - 5.41)	.001
	**Victims of violence at T3**			n.s.
	No	549	1.00	
	Yes	350	1.22 (0.94 - 1.59)	n.s.
	**Age**	-	0.99 (0.91 – 1.07)	n.s.
	**Access condoms at T3**	-	1.32 (1.14 - 1.53)	.001
	**SES at T3**	-	1.32 (1.24 - 1.53)	.001
	**Communication at T2**	-	1.20 (1.03 - 1.40)	.05
Nagelkerke’s pseudo R^2^ =.275				

Three multiple ordinal logistic regression analyses with control for cluster effects.

The strongest predictor in each of the models was condom use at the first of the two relevant measurement occasions. The odds of condom use increased with being female, socioeconomic status (significant for two of the three models), and access to condoms. Being exposed to violence was not associated with condom use at any occasion. Communication (global sumscore) predicted consistent condom use prospectively, and significance was obtained in all three models. The odds ratios were 1.22 (T1-T2), 1.21 (T1-T3) and 1.20 (T2-T3). This corresponds to the following odds ratios for consistent users, when taking never-users as the comparison group: 1.49, 1.46, and 1.44. It is also worth noting that the proportion reporting consistent condom use is lowest in Dar es Salaam, higher in Mankweng, and highest in Cape Town.

Analyses identical to the ones presented in Table 4 were carried out for males and females separately. The odds ratio values for communication at first occasion for males varied from 1.18 to 1.28 and for females from 1.11 to 1.24. The differences between males and females with regard to prospective prediction of condom use were not close to being significant.

In a series of 18 multiple ordinal logistic analyses (tables not shown), the global sumscores were replaced with more specific sumscores (one for each communication partner across topics and one for each topic across communication partners) one by one. Odds ratio values varied between 1.10 and 1.23 and were statistically significant (p<.05) in fifteen cases, borderline significant (p<.10) in two cases and not significant (p=.15) in one. There was no clear indication that communication with one particular category of adults (parents, other family members or teachers) was more strongly associated with condom use than communication with the other categories. And there was no strong indication that communication on one particular topic (HIV/AIDS, abstinence or condoms) was more strongly associated with condom use than communication on any of the other topics.

In another series of ordinal logistic regression analyses (tables not shown), communication at the current occasion (T2 for the first analysis and T3 for the second and third analyses, similar to the three analyses presented in Table 4) was added to the models. This reduced the odds of the communication predictor at the previous occasion, indicating that at least part of the prediction of condom use by previous communication was mediated by communication at the most recent occasion.

Additional findings worth mentioning relate to gender and socioeconomic status. Condom use and consistent condom use was more common among females. Even after controlling for a number of confounders, the odds ratio values when predicting condom use from gender (males as reference group) varied between 1.79 and 1.96 (all significant). High scores on our socioeconomic status index were associated with higher odds of using condoms (two out of three associations were significant).

## Discussion

The communication scales (subscales as well as total scale) had high internal consistency (alpha coefficients .83 and higher), thus indicating that we were able to measure communication about HIV/AIDS, abstinence and condoms constructs across three communication partners and on three occasions with an acceptable degree of accuracy. The correlations among communication sumscores across measurement occasions are sufficiently high to demonstrate some stability in such communication. The patterns are also consistent with the assumption that longer time spans produce lower autocorrelations. These findings give reasons for being confident about the quality of the communication scale.

In line with findings from some previous studies [10-14,19,20,22], the present study shows significant cross-sectional associations between communication variables and condom use. The associations are quite similar across communication partners (parents, other adult family members, and teachers) and topics discussed (HIV/AIDS, abstinence and condom – tables not shown). Communication sumscore means are generally highest among those who use condoms consistently, lower among the occasional condom users, and lowest among the never-users. To the best of our knowledge, this is the first time such a broad and consistent pattern of cross-sectional associations between communication variables and condom use has been demonstrated in a study from sub-Saharan Africa.

We have only identified one previous study which examines the association between communication on sexuality issues and condom use prospectively [18]. They found some prospective association between degree of comfort in talking with parents about sex and condom use. In our study similar associations proved significant in three different statistical analyses with control for a number of other predictors. This is consistent with the idea that communication between adolescents and responsible adults leads to safer sexual practices. However, there are other possible explanations. There could be third variables, not measured in our study, which accounted for the association. Prospective longitudinal designs cannot completely rule out such alternative explanations. However, since we controlled for condom use at the previous data collection occasion as well as a number of possible confounders in each model, we have at least moved one step towards confirming causality. An important next step in this line of research would be to demonstrate that experimentally-induced increases in communication would be followed by increased condom use.

We have shown that communication predicts condom use prospectively over six and even twelve months. Odds ratio values ranging from 1.20 to 1.22 amount to odds ratio values ranging from 4.29 to 4.91 if we compare the highest with the lowest value on the communication scale and if we compare consistent use with never-use. If we had measured the extent of high quality communication on sexuality issues, the prediction would certainly have been stronger. High quality communication in this context could include openness, dialogue rather than monologue, contextualisation and specificity of the messages, timing of communication, and a context characterized by authoritative rather than authoritarian parenting [32-34].

Our review of previous studies, as well as the one by Markham and associates [23] did not provide much consistent evidence for a positive association between adolescent-adult communication and condom use. Still, the findings from the present study are highly consistent. The more adolescents communicate on sexuality issues with parents, other adult family members and teachers, the higher are the odds of using condoms and using condoms consistently. One reason why we can draw such a consistent picture is that the present study is based on a high number of observations. Our estimates are therefore fairly accurate. Furthermore we may hypothesize that the communication skills may be higher and the context of communication taking place may be more favourable in families where there is at least some communication on sexuality issues. Our simple and straightforward measures of communication may reflect not only amount of communication, but also other aspects such as quality and context.

Gender differences in sexuality communication have been focussed in a number of studies among adolescents in sub-Saharan Africa [9,31,35]. No study has, however, examined gender differences in the prospective association between sexuality communication and condom use. If male dominance in sexual relationships was pronounced, one might hypothesize that the association was stronger for males than for females. In the present study the interaction with gender was, however not close to being significant. Perhaps adolescent girls in the sub-Saharan African contexts covered by this study after all do have some influence on young couple’s decisions regarding use of condoms.

It is well established that young males more than females tend to take risk, and this has also been shown to be the case for condom use [36]. Consistent with this, we found condom use and consistent condom use to be more common among females than among males. In some South African studies condom use has been shown to be more prevalent among males than among females [37]. Factors that may contribute to explaining these apparent inconsistencies deserve to be examined in future studies.

Previous studies from South Africa have shown that condom use is less common among disadvantaged groups [38,39]. This pattern is confirmed in the present study. High scores on our socioeconomic status index are associated with condom use. Interventions specifically targeting disadvantaged groups should be developed and tested. Action to reduce socioeconomic inequalities and improve the situation of disadvantaged groups may, however, prove to be the most effective remedy.

### Strengths and limitations of the present study

Local personnel and experts, familiar with the local languages and cultures, were involved in the development of instruments for the present study. Most questions and scales used for data collections were carefully piloted and tested. The internal consistency of scales for the measurement of interpersonal communication is high. The number of observations in this study is also quite high. Cluster effects have been systematically adjusted for in all statistical analyses of data. The findings are highly consistent. These are obvious strengths of this study. However, one clear limitation is in the measurement of communication between significant adults and adolescents on sexuality related issues. Besides the frequency of communication, which was measured in this study, additional information on the characteristics of the communication (dialogue, monologue, structured or structured, perceived quality of the communication, and timing among others) could have improved our prediction of condom use.

Another possible limitation of this study is also related to the way communication with adults is measured. The very first item was phrased like this: ‘How often do your parents or guardians talk with you about HIV/AIDS?’ The phrase ‘talk with you’ is used consistently for all nine items in the communication scale, and could imply that we refer to communication initiated by adults. In the light of Kerr & Stattin’s view that spontaneous disclosure of information is more important than parental monitoring in predicting adolescents’ adjustment [9], this way of phrasing the items may not be the best one. In future studies we recommend that other ways of wording such items are tested, for instance: ‘How often do you and your parents or guardians talk about HIV/AIDS?’ This is more neutral and does not imply that the parents initiate the communication. There could also be separate questions specifically related to the issue of who initiates such communication.

In spite of the rather simple and straightforward way communication is measured in this study, we have been able to show that communication predicts condom use prospectively. It is likely that high quality communication and communication containing specific messages and advice from adults would show stronger prediction of condom use than what has been shown in the present study. Separate measurement of communication with father and mother (or male and female caregivers) would also have added to the richness of the data. Communication on sexuality with fathers and mothers may function differently, especially for boys vs. girls. In future studies communication with father and mother should be measured separately.

## Conclusion

On the basis of our findings, we conclude that adolescent communication with adults on sexuality related issues prospectively predicts condom use, including consistent condom use, among high school adolescents significantly in all three analyses presented. The current study brings forth new evidence in support of the assertion that promoting communication on sexuality and related issues between adolescents and responsible adults may contribute to increasing the likelihood of condom use among adolescents. Sexuality communication that is initiated already before or at the time of sexual debut may increase the likelihood of adolescents enacting safe sex practices such as consistent condom use. Future interventions and programmes for promotion of healthy sexual behaviour should, however, not only focus on increasing the amount of such communication, but also on improving its quality as well as other aspects of adolescents’ relationship with parents, teachers and other adults that are important to them [11,14].

There is an obvious need to train parents in sub-Saharan contexts such as the ones covered by this study in communicating on sexuality with their adolescent children. Not all parents have the skills and the confidence needed to initiate such discussion. Training programmes should therefore not only contribute to higher awareness and better knowledge among parents, but also more generally to improve their communication- and parenting skills. There is a lot to learn from decades of research on parenting and adolescent-parent interaction [3]. Programmes aiming at improving parenting skills and skills to communicate on sexuality with adolescent children should be research based, and evaluation studies should utilize strong experimental designs. Experimental studies of parent-offspring communication on sexuality and its behavioural effects among adolescents would contribute to throwing more light on the possible causal mechanisms involved. And even more important, such studies could contribute to informing intervention programmes aimed at reducing the risk that young people in sub-Saharan African countries and contexts get infected with sexually transmitted diseases.

## Competing interests

The authors declare that they have no competing interests.

## Authors’ contributions

FSN contributed to the coordination of the study, analysed data for the present article, and wrote most of the manuscript. LEA was the coordinator of the SATZ project and supervised data analysis and writing of the present publication. HEO, SK and CM contributed to designing the study, they planned and administered data collections and computerizing of data in their respective sites, commented on all drafts of the manuscript and provided site-specific information relevant to understanding findings. AW contributed to the coordination of the study including quality control of data and merging of data files. She also commented on all drafts of the manuscript. All authors read and approved the final manuscript.

## Pre-publication history

The pre-publication history for this paper can be accessed here:

http://www.biomedcentral.com/1471-2458/13/874/prepub
